# Association between Attention Deficit Hyperactivity Disorder and Suicide Attempts in Patients with Bipolar Disorder

**Published:** 2019-07

**Authors:** Jafar Fili, Marzieh Nojomi, Katayoon Razjouyan, Mojgan Kahdemi, Rozita Davari-Ashtiani

**Affiliations:** 1 Department of Psychiatry, Shafa Hospital, Guilan University of Medical Sciences, Rasht, Iran.; 2 Preventive Medicine & Public Health Research Center, Iran University of Medical Sciences, Tehran, Iran.; 3 Department of Psychiatry, Imam Hossein Hospital, Shahid Beheshti University of Medical Sciences, Tehran, Iran; Behavioral Sciences Research Center, Shahid Beheshti University of Medical Sciences, Tehran, Iran.

**Keywords:** *Attention Deficit Hyperactivity Disorder*, *Bipolar Disorder*, *Suicide*

## Abstract

**Objective:** The present study aimed to examine the association between ADHD and suicide attempts among adolescents with bipolar disorder.

**Method** **:** Participants were 168 adolescents who fulfilled DSM-IV-TR criteria for bipolar disorder. They were divided into 2 groups: The first group of patients with bipolar disorder with a history of suicide attempts (n = 84) and the second group without a history of suicide attempts (n = 84). ADHD and other variables were analyzed using a chi-squared test and logistic regression model.

**Results: **No significant difference was observed between the 2 groups in comorbidity of ADHD and other psychiatric disorders (P > 0/05). In the logistic regression model, and after controlling for other factors, gender (OR = 3.9, CI 95%: 1.5-9.6) and history of sexual abuse (OR = 3.4; CI 95%: 1.06-11.3) were the only 2 factors associated with a history of suicide attempts.

**Conclusion: **No significant association was found between ADHD and suicide attempts in adolescents with bipolar disorder.

The prevalence of bipolar disorder among children and adolescents has been reported to be 6.7% (1). Bipolar disorder is a severe disorder in childhood and it continues into adulthood with an increased risk of suicide (2). Of patients with bipolar disorder, 25%-50% have attempted suicide at least once and around 15% have committed suicide (3). Attention-deficit/ hyperactivity disorder (ADHD), anxiety disorders, substance use disorders, and disruptive behavior disorders are the most common comorbid disorders in patients with bipolar disorder (4). ADHD is one of the most frequent neurodevelopmental disorders in childhood that is likely to exist before the incidence of bipolar disorder or simultaneously with bipolar disorder (4, 5). ADHD with an onset prior to bipolar disorder can be found in up to 90% of prepubertal children and in almost half of adolescents with bipolar disorder. Suicidal behaviors are a common problem among patients with ADHD, which may lead to negative social consequences (6).

The rate of suicide attempts and completed suicide increases linearly from childhood to adulthood (7). Among the general US population, in every 30 attempts, one results in completed suicide, while the ratio for patients with bipolar disorder is 1 in every 3-4 attempts (8). Previous studies have demonstrated that the overwhelming majority, nearly 96%, of adolescents with a history of suicide attempts have at least 1 psychiatric disorder (7). Despite a common comorbidity between ADHD and bipolar disorder (4, 5), few studies have been conducted on the effect of ADHD on suicide attempts among patients suffering from bipolar disorder. Among 15-to-24-year-old patients with bipolar disorder, ADHD has been reported to be an independent risk factor for suicide attempts (9).

The present study was conducted to study the association between ADHD and suicide attempts among adolescents suffering from bipolar disorder.

## Materials and Methods

This was a case control study for which ethical approval was obtained from the Ethics Committee of Shahid Beheshti University of Medical Sciences. The scope of the study was explained to the patients at the beginning and confidentiality of information was ensured. All participants provided oral and written informed consent before the study. Participants were consecutively selected from 2 university hospitals. All patients aged 12-18 years who were admitted to these 2 hospitals and received primary diagnosis of bipolar disorder were enrolled. Data collection began in April 2016. A total of 168 patients were studied and data collection was completed at the end of May 2017.

The patients were divided into 2 groups: the case group (patients with bipolar disorder with a history of suicide attempts) and the control group (patients with bipolar disorder without a history of suicide attempts). The history of suicide attempt was based on the interview with the patients and their parents considering 3 criteria: (1) A behavior that is potentially harmful to the individual, which he does so to die. (2) There is evidence of this behavior or the existing conditions. (3) The suicide attempt is likely to result in a serious injury (10). Given the limitation of statistical data and the 20% difference (based on the consensus of experts) between the 2 groups studied, the sample size was estimated to be 84 individuals per group with a power of 80% and error of 5%. All inpatient adolescents in the psychiatric wards of these 2 hospitals were visited by the researcher. Demographic information of the patients and their families was extracted by conducting interviews with the adolescents and their parents. Diagnosis of bipolar disorder and the comorbidities was confirmed by a child and adolescent psychiatrist based on the Persian version of the Kiddie Schedule for Affective Disorders and Schizophrenia‐Present and Lifetime Version (K‐SADS‐PL‐P), which is a semi-structured interview whose reliability and validity has been studied in Iran (11). History of abuse was also obtained based on the interview with the patient. The inclusion criteria allowed all 12-to-18-year-old patients suffering from bipolar disorder based on K-SADS-PL-P to enter the study. The exclusion criteria dismissed all patients with intellectual disability, autism spectrum disorder, conduct disorder, substance use disorder, and non-psychiatric chronic diseases, such as epilepsy. 

***Statistical Analysis***

SPSS version 19 was used to describe and analyze data. To compare the variables across the 2 groups, a chi-squared test was used. Statistical significance was set at 0.05. Also, to assess the independent association between suicide attempts and covariates, a logistic regression analysis was used. The dependent variable was history of suicide attempts in patients with bipolar disorder. 

## Results

Results of the 168 patients, 103 were female and 65 male. The mean age of patients in the case and control groups was 15.5 and 15.3, respectively. There was a significant difference in gender. The sociodemographic characteristics of patients are shown in Table 1. The participants did not receive any treatment for ADHD during hospitalization.

In contrast to the frequency of physical abuse, the frequency of sexual abuse was significantly higher among participants with bipolar disorder and a history of suicide attempts. With respect to the frequency of psychiatric disorders, the highest frequency was related to ADHD in both groups. The 2 groups were not significantly different in the frequency of comorbid psychiatric disorders (Table 2).

The frequency of psychotic symptoms in the case group was lower than in the control group (14.3% vs 25%). Participants were studied to find the frequency of different types of bipolar disorder (type I, II, and NOS). Bipolar I disorder was the most frequent and bipolar II disorder was the least frequent; however, no significant difference was observed between the 2 groups (Table 2). The variables closely related to history of suicide in the 2-variable analysis were analyzed in the logistic model, and the only significant suicide-related factors were still “female gender” (OR= 3.9, CI 95%: 1.5-9.6) and “history of sexual abuse” (OR= 3.4; CI 95%: 1.06-11.3) (independent relationship). Other factors did not show a significant relationship with suicide attempts (Table 3).

**Table 1:** Sociodemographic Characteristics of Patients with and without Bipolar Disorder

		**Bipolar disorder ** **with suicide ** **attempts(N=84)**	**Bipolar disorder ** **without suicide ** **attempts(N=84)**	**P value**
		**Number (%)**	**Number (%)**	
Age	12-14	17(20)	25(29.8)	0.154
	15-18	67(79.8)	59(70)	
Gender	Boy	22(33.8)	43(66.2)	0.001
	Girl	62(60.2)	41(39.8)	
Number of children	Only child	14(16.7)	17(20.2)	0.551
	Multiple children	70(83.3)	76(79.8)	
Living with parents	With both	55(65.5)	62(73.8)	0.193
	With one parent	22(26.2)	20(23.8)	
	Without parents	7(8.3)	2(2.4)	
History of abuse	Physical	18(21.4)	18(21.4)	0.005
	Sexual	19(22.6)	6(7.1)	
Family’s economic status	Low	16(19)	25(8.29)	0.056
	Average	60(71.4)	45(6.53)	
	High	8(9.5)	14(7.16)	
History of psychiatric disorder (mother)	yes	62(73.8)	53(63.1)	0.135
History of psychiatric disorder (father)	yes	63(75)	54(64.3)	0.131
History of psychiatric disorder (siblings)	yes	50(59.5)	39(46.4)	0.089
History of psychiatric disorder (relatives)	yes	62(73.8)	64(76.2)	0.177
History of suicide attempts (mother)	yes	10(11.9)	8(9.5)	0.32
History of suicide attempts (father)	yes	5(6)	2(2.4)	0.117
History of suicide attempts (parents)	yes	13(56.5)	10(43.5)	0.183
History of suicide attempts (siblings)	yes	10(11.9)	1(1.2)	0.047
History of suicide attempts (relatives)	yes	21(25)	14(16.7)	0.033

**Table 2:** Comorbid Psychiatric Disorder in Bipolar Disorder with and without Suicide Attempts

	**Bipolar disorder with ** **suicide attempts (N=84)**	**Bipolar disorder without ** **suicide attempts (N=84)**	**P ** **value**
	**Number (%)**	**Number (%)**	
Attention deficit hyperactivity disorder	59(70)	47(56)	0.055
Obsessive compulsive disorder	23(27.4)	32(38.1)	0.139
Oppositional defiant disorder	24(28.6)	26(31)	0.736
Anxiety disorder	34(40.5)	29(34.5)	0.426
Bipolar disorder with psychotic features	12(14.3)	21(25)	0.081
Bipolar disorder Type 1 Type 2 NOS	41(34.4) 8(6.7) 35(29.4)	43(36.2) 6(5) 30(29.4)	0.45

**Table 3:** Logistic Model for Factors Associated With a History of Suicide Attempts in Bipolar Disorder

	**OR(95%CI)**	**P value**
Gender	3.908 (1.580-9.665)	0.003
Low family economic level	0.798 (0.188-3.379)	0.415
Attention deficit hyperactivity disorder	1.792 (0.742-4.331)	0.195
Suicide attempts in second-degree relatives	2.182 (0.783-6.080)	0.136
Suicide attempts in siblings	6.663 (0.715-62.06)	0.096
History of physical abuse	1.123 (0.357-3.53)	0.843
History of sexual abuse	3.469 (1.065-11.306)	0.039
Constant	0.032	0

## Discussion

This was the first study in Iran to estimate the association between ADHD and suicide attempts in adolescents with bipolar disorder. The results of this study showed that, although the frequency of ADHD was higher in patients with bipolar disorder and a history of suicide attempts compared to patients with bipolar disorder and without a history of suicide attempts (a 14% difference), the difference was not significant. Furthermore, female gender and history of sexual abuse had an independent association with suicide attempts in patients with bipolar disorders. However, there are always some limitations in obtaining history of abuse, as some adolescents may not report it. Other covariates did not show any significant association with suicide attempts in patients with bipolar disorders.

Results of this study are comparable with those of Goldstein et al and Algorta. Goldstein et al indicated that the frequency of ADHD was lower in the group of patients suffering from bipolar disorder with a history of suicide than that of the group of patients suffering from bipolar disorder without a history of suicide (12). Also, Algorta et al demonstrated that the frequency of ADHD, oppositional defiant disorder, and anxiety disorder was not significantly different in the 2 groups of patients suffering from bipolar disorder with and without a history of suicide (13). Findings of this study are consistent with those of previous studies confirming that the frequency of ADHD was not significantly different in the 2 groups of patients suffering from bipolar disorder with and without a history of suicide attempts. Contrary to the present study, Lan et al indicated that ADHD is an independent risk factor among adolescents and adults with bipolar disorder (9). This may be due to the fact that Lan et al examined patients aged 15-24 years, with a mean age of 19, while the present study was conducted exclusively on youths aged 12-18 years, with a mean age of 15.5. 

According to previous studies, impulsivity, as a symptom in ADHD, is associated with increased suicide attempts (14, 15). However, in the present study, no significant difference was observed between the 2 groups with respect to the frequency of ADHD. According to Eaton et al, female gender is a risk factor for suicide attempt (16). In the present study, suicide attempts in patients with bipolar disorder were significantly higher in girls than in boys, which is in line with previous studies (17-19). Nevertheless, one should be cautious in generalizing the results, as this study was conducted on patients with bipolar disorder who needed to be admitted and the sample might have had some peculiarities, not fully representing all patients with bipolar disorder. 

## Limitations

There are some limitations in this study which should be noted. First, the number of girls in the case group was significantly higher than in the control group. Thus, as ADHD is more frequent in boys, no difference was found between boys and girls, as there were more girls in the case group. Therefore, confounders were adjusted in the logistic regression analysis (Table 3). Second, most of the patients had been under pharmacological treatment before their hospitalization. Thus, this factor might have affected their suicide attempts either directly or indirectly. However, this has not been taken into account in the present study. Third, in spite of the factors evaluated in this study, other factors influencing suicide attempts, such as severity of psychiatric disorder and environmental stressors, were not evaluated. It was attempted though to minimize these limitations through adopting the principle of confidentiality and having a control group. Forth, the exact history of drug treatment for ADHD was not known at the time of the patients' past suicide attempts, which can be another limitation of this study. Finally, the study was conducted on inpatients with bipolar disorder; therefore, caution should be taken in generalizing the results to all individuals with bipolar disorder. Conducting further research in other settings using more precise methodology is highly recommended. 

Because of the high frequency of suicide attempts among patients with bipolar disorder, they should be carefully assessed for suicidality.

## Conclusion

According to the finding of this study no significant association was found between ADHD and suicide attempts in adolescents with bipolar disorder, but more studies are needed to evaluate this relationship by including larger sample with inpatient and outpatient participants. 
